# Binding of von Willebrand Factor to Complement C1q Decreases the Phagocytosis of Cholesterol Crystals and Subsequent IL-1 Secretion in Macrophages

**DOI:** 10.3389/fimmu.2019.02712

**Published:** 2019-11-21

**Authors:** Claudia Donat, Sophia Thanei, Marten Trendelenburg

**Affiliations:** ^1^Laboratory of Clinical Immunology, Department of Biomedicine, University of Basel, Basel, Switzerland; ^2^Division of Internal Medicine, University Hospital of Basel, Basel, Switzerland

**Keywords:** macrophages, complement C1q, von Willebrand factor, cholesterol, atherosclerosis, innate immunity

## Abstract

Complement C1q, the initiation molecule of the classical pathway, exerts various immunomodulatory functions independent of complement activation. Non-classical functions of C1q include the clearance of apoptotic cells and cholesterol crystals (CC), as well as the modulation of cytokine secretion by immune cells such as macrophages. Moreover, C1q has been shown to act as a binding partner for von Willebrand factor (vWF), initiation molecule of primary hemostasis. However, the consequences of this C1q-vWF interaction on the phagocytosis of CC by macrophages has remained elusive until now. Here, we used CC-C1q-vWF complexes to study immunological effects on human monocyte-derived macrophages (HMDMs). HMDMs were investigated by analyzing surface receptor expression, phagocytosis of CC complexes, cytokine secretion, and caspase-1 activity. We found that vWF only bound to CC in a C1q-dependent manner. Exposure of macrophages to CC-C1q-vWF complexes resulted in an upregulated expression of phagocytosis-mediating receptors MerTK, LRP-1, and SR-A1 as well as CD14, LAIR1, and PD-L1 when compared to CC-C1q without vWF, whereas phagocytosis of CC-C1q complexes was hampered in the presence of vWF. In addition, we observed a diminished caspase-1 activation and subsequent reduction in pro-inflammatory IL-1β cytokine secretion, IL-1β/IL-1RA ratio and IL-1α/IL-1RA ratio. In conclusion, our results demonstrate that vWF binding to C1q substantially modulates the effects of C1q on HMDMs. In this way, the C1q-vWF interaction might be beneficial in dampening inflammation, e.g., in the context of atherosclerosis.

## Introduction

The complement system is a highly effective part of the innate immune system. The multiple functions of complement include defense against bacterial infections, bridging innate and adaptive immunity and the clearance of immune complexes, and components of inflammation (1). The complement system can be activated through three distinct pathways: the classical, the lectin and the alternative pathway. All three pathways converge in a shared terminal response resulting in the formation of C5a and C3a as potent inflammatory effector molecules and C5b-9 as membrane attack complex. However, each pathway is initiated through different characteristic recognition molecules (2). The initiation of the classical pathway is triggered by C1q through sensing of bound antibodies as well as pathogen- and damage-associated molecular patterns (PAMPs/DAMPs). In addition, more recent research has shown a number of functions for C1q that are independent of downstream complement activation (3). On the one hand, opsonization with C1q enhances the clearance of diverse structures, namely immune complexes (4) and apoptotic cells (5) as well as atherogenic lipoproteins (6) and cholesterol crystals (CC) (7) by phagocytes. On the other hand, anti-inflammatory properties for C1q have been well-described. For example, bound C1q decreases the release of pro-inflammatory cytokines and increases the production of anti-inflammatory mediators by phagocytes (8, 9). Additionally, the presence of C1q on apoptotic cells skews macrophage polarization toward an anti-inflammatory phenotype (10).

Apart from C1q's extensively studied involvement in immunity, a complex cross-talk between complement and coagulation is becoming more and more evident (11). Complement components have been found to induce hemostasis and *vice versa* coagulation factors can trigger complement activation, thereby combining two powerful plasma cascades. Within the hemostatic cascade, von Willebrand factor (vWF) acts as an important starter molecule by mediating platelet adhesion and aggregation. Immune cells, such as macrophages, are competent to take up and clear vWF through scavenger receptors (12, 13). Moreover, vWF has been shown to interact with complement factor H (14, 15) and therefore can modulate the activation of complement via the alternative pathway (16). Furthermore, a direct interaction between vWF and C1q was found by our group, demonstrating that C1q, bound to surfaces such as apoptotic cells, acts as a binding partner for vWF (17). The C1q-vWF interaction also seems to occur on the surface of CC.

CC can be found as a characteristic feature in the intima of atherosclerotic arteries from early lesions to late plaque (18) and are widely used in *in vitro* models of atherosclerosis (19–21). Formation of CC occurs upon fatty streak development by an increased uptake and exhausted efflux of cholesterol by lipid-laden macrophages known as foam cells. In *in vitro* and *in vivo* models of atherosclerosis, CC have been implicated in the activation of the NOD [nucleotide oligomerization domain]-, LRR [leucine-rich repeat]-, and PYD [pyrin domain]-containing protein 3 (NLRP3) inflammasome and downstream cytokine secretion, consequently triggering local and systemic inflammation (22–24). While the role of CC and macrophages in atherosclerosis appears unambiguous, C1q can play a dual role. On the one hand, C1q bound to oxidized low-density lipoproteins (LDL) or CC has been shown to activate the classical pathway, and in this context to drive the progression of atherosclerosis in animal models (25, 26). On the other hand, C1q has also been described to be protective in early atherosclerosis *in vivo* (27, 28) and to increase cholesterol efflux transporter expression *in vitro* (6), suggesting atheroprotective properties. Similarly, the role of vWF in atherosclerosis is still a matter of debate. Although various studies suggest that vWF deficiency provides protection from atherosclerosis in animals, in humans, an unequivocal protective effect of vWF deficiency on atherosclerosis has not been demonstrated so far (29).

In summary, CC, macrophages, C1q and vWF have all been implicated in atherosclerosis. Nevertheless, the consequences of the interaction between C1q and vWF, especially on phagocytes, remain to be determined. In order to better understand this interaction, the aim of our study was to investigate the immunological effect of complexes consisting of cholesterol crystals, C1q and von Willebrand factor (CC-C1q-vWF complexes) by studying receptor expression, phagocytosis and cytokine secretion of macrophages.

## Materials and Methods

### Preparation of CC

Cholesterol (suitable for cell culture, Sigma Aldrich, St. Louis, MO, USA) was dissolved in 95% ethanol at 60°C (12.5 g/l), sterile filtered and allowed to crystallize at room temperature (RT) for 7 days (d). Excess liquid was removed from the suspension, followed by drying for 5 d. Finally, CC were ground and stored as stock CC at −20°C until use.

### Preparation of CC, CC-C1q, and CC-C1q-vWF Complexes for Characterization of C1q and vWF Binding

Dry stock CC were weighed and suspended in PBS (Life Technology, Carlsbad, CA, USA) at a concentration of 1.6 mg/ml, vortexed, and sonicated until a visually homogenous suspension was achieved. This CC suspension was split in three fractions for generation of CC, CC-C1q complexes, and CC-C1q-vWF complexes. Fractions were washed with PBS by centrifugation (1,000 x g, 5 min, RT) and resuspended at the same concentration. For generation of CC-C1q complexes, 50 μg/ml purified C1q (Complement Technology, Tyler, Tx, USA), diluted in PBS, was added and incubated for 1 h at RT on a shaker (700 rpm). Afterwards, CC and CC-C1q complexes were washed (as described above). For generation of CC-C1q-vWF complexes, 10 μg/ml recombinant vWF {provided by Baxalta, Lexington, MA, USA [former Baxter; characterization by Turecek et al. (30)]}, diluted in PBS, was added to washed CC-C1q complexes, vortexed rigorously, and further incubated for 1 h at RT on a shaker (700 rpm). After another washing step, CC complexes were further incubated with monoclonal mouse anti-C1q [clone 32A6 cell supernatant (31)], diluted 1:20 in PBS, or polyclonal rabbit anti-vWF (Abcam, Cambridge, UK), diluted 1:1,000 in PBS, for 1 h at RT on a shaker (700 rpm). Secondary antibody staining was performed with donkey anti-mouse IgG-AlexaFluor (AF)555 (Life Technology) and goat anti-rabbit IgG-AF647 (Abcam), both diluted 1:200 in PBS/1%BSA (Sigma Aldrich)/0.5 M NaCl for 30 min at 4°C in the dark, followed by a final wash step and resuspension in PBS/1%BSA/0.5 M NaCl. All fractions were washed and treated with either active substance (protein or antibodies) or solution only in the same manner. For flow cytometry, data were acquired using a BD Accuri 6 (BD Biosciences, San Jose, CA, USA) and analyzed with FlowJo10. For confocal microcopy, CC were spun onto cytoslides (Shandon, Pittsburg, PA, USA) by a Cytospin centrifuge (Thermo Fisher Scientific, Waltham, MA, USA) and analyzed using Nikon A1R Nala and NIS software (both Nikon, Tokyo, Japan). For imaging flow cytometry, analyses were carried out using ImageStreamX Mark II and IDEAS software (both EMD Millipore, Billerica, MA, USA).

### Cell Culture

Peripheral blood mononuclear cells were isolated from fresh buffy coats (Blood Transfusion Center of the University Hospital Basel, Basel, Switzerland) by density gradient centrifugation using Lymphoprep (Stemcell Technologies, Vancouver, Canada). Monocytes were obtained by CD14+ magnetic-activated cell separation beads (Miltenyi, Bergisch Gladbach, Germany) according to the manufacturer's instructions (yielding an average purity of 95–98% CD14+ monocytes determined by flow cytometry). Monocytes were differentiated into human monocyte-derived macrophages (HMDMs), cultured in DMEM supplemented with 100 U/ml penicillin and 100 μg/ml streptomycin (DMEM+), 10% fetal calf serum (FCS) (all from Life Technology), and 50 ng/ml GM-CSF (Immunotools, Frisoythe, Germany) at a cell concentration of 5 × 10^5^ cells/ml in 6-well plates (BD Biosciences, Franklin Lakes, NJ, USA) and maintained in 5% CO_2_ at 37°C for 7 days.

### Treatment With CC Complexes

After 7 days, HMDMs were washed with prewarmed DMEM+, optionally stimulated with 100 ng/ml lipopolysaccharide (LPS) (*E. coli* O127:B8, Sigma Aldrich), diluted in prewarmed DMEM+, and treated with CC, CC-C1q, or CC-C1q-vWF complexes for indicated time points. CC and CC-complexes were prepared as described above, washed once with PBS by centrifugation (1,000 × g, 5 min, RT) and resuspended in prewarmed DMEM+ at a final concentration of 0.5 mg/ml before adding to cells.

### Surface Receptor Expression

HMDMs were stimulated with LPS and treated with CC, CC-C1q, or CC-C1q-vWF complexes as described above. After 18 h, HMDMs were washed with PBS and incubated with PBS/10 mM EDTA (AppliChem, Darmstadt, Germany) for 30 min at 4°C. Cells were collected in FACS buffer (PBS/0.1% FCS/1 mM EDTA) and resuspended at a cell concentration of 5 × 10^5^ cells/100 μl and incubated with 2 μg/ml of human IgG for 45 min at 4°C to block unspecific binding of antibodies to Fcγ receptors. Staining was performed for 30 min at 4°C in the dark in PBS using the following antibodies: anti-MHC II-FITC (Immunotools), anti-tyrosine-protein-kinase Mer (MerTK)-PE (R&D Systems, Minneapolis, MN, USA), anti-programmed death ligand 1 (PD-L1/CD274)-APC and anti-CD14-PeCy7 (both from Biolegend, San Diego, CA, USA) (antibody panel 1), or anti-CD86-FITC (Biolegend), anti-lipoprotein receptor-related protein 1 (LRP-1/CD91)-PE (Thermo Fisher Scientific, Waltham, MA, USA), anti-leukocyte-associated immunoglobulin-like receptor 1 (LAIR1/CD305)-AF647 and anti-scavenger receptor A 1 (SR-A1/CD204)-PeCy7 (both from Biolegend) (antibody panel 2). HMDMs were washed and resuspended in FACS buffer. Data were aquired using a BD LSRFortessa (BD Biosciences) and analyzed with FlowJo10. Gating was performed on SSC/CD14+ cells (antibody panel 1) or SSC/CD91+ cells (antibody panel 2), respectively, and geometric mean fluorescence intensity (gMFI) was calculated.

### Phagocytosis Assay

#### Assessment of Granularity of HMDMs

HMDMs were kept untreated or treated with CC, CC-C1q or CC-C1q-vWF complexes as described above. After 18 h, HMDMs were harvested with PBS/10 mM EDTA and resuspended at a cell concentration of 5 × 10^5^ cells/100 μl in FACS buffer. HMDMs were stained with anti-CD11c-APC (Biolegend) for 30 min at 4°C in the dark. Data were aquired using a BD LSRFortessa (BD Biosciences) and analyzed with FlowJo10. For the quantification of phagocytosis the percentage of CD11c+ cells with high cell granularity, indicated by a shift into the side scatter (SSC) ^high^ gate (gate set according to shift in SSC from CD11c+ untreated to CC treated cells), was determined.

#### Assessment of Phagocytosed pHrodo-Dyed CC Complexes

HMDMs were harvested with PBS/10 mM EDTA and resuspended in phagocytosis buffer (DMEM+/12.5 mM HEPES (Sigma Aldrich)/5 mM MgCl_2_) at a density of 5 × 10^5^ cells/100 μl. For pHrodo-dyed CC complexes, 1 mg/ml CC were suspended in 0.1 M NaHCO_3_ buffer (pH 8.3) and incubated with 10 μg/ml pHrodo Red Ester (Thermo Fisher Scientific) for 1 h in the dark before the addition of C1q or C1q-vWF as described above. After a final wash, pHrodo-dyed CC complexes were added to HMDMs at a concentration of 0.5 mg/ml and incubated at 37°C for 30 min. Unphagocytosed CC complexes were washed away and HMDMs were stained with anti-CD11c-FITC (Bio-Rad, Hercules, CA, USA) for 30 min at 4°C in the dark and resuspended in FACS buffer. Data were aquired using a Beckman Coulter CytoFLEX (Beckman Coulter, Brea, CA, USA) and analyzed with FlowJo10. For the quantification of phagocytosis, the percentage of CD11c+ cells with a shift into the pHrodo Red Ester+ gate (gate set according to shift in pHrodo Red Ester from CD11c+ untreated to CC treated cells) was determined.

### Quantification of Secreted Cytokine Levels

HMDMs were stimulated with LPS and treated with CC, CC-C1q, or CC-C1q-vWF complexes as described above. After 18 h, supernatants were collected, centrifuged to remove cellular debris and CC and stored at −80°C until measurement. Analyses of cytokine secretion were carried out in duplicates with ELISA kits according to the manufacturer's instructions. IL-1ß, IL-1α, IL-6, and IL-10 were measured using Biolegend ELISA kits, IL-18 and IL-1RA using Abcam ELISA kits and TNFα using a BD Bioscience ELISA kit.

### Caspase-1 Activity Assay

HMDMs were kept untreated or treated with CC, CC-C1q, or CC-C1q-vWF complexes as described above. After 18 h, HMDMs were harvested with PBS/10 mM EDTA and resuspended at a cell concentration of 5 × 10^5^ cells/ml. Cells were incubated for 1 h with fluorochrome-labeled inhibitors of caspases (FLICA) probes for caspase-1 detection according to the manufacturer's instruction (FAM FLICA Caspase-1 Assay Kit, Immunochemistry Technology, Bloomington, MN, USA). HMDMs were stained with anti-CD11c-APC (Biolegend) for 30 min at 4°C in the dark. Data were aquired using a BD LSRFortessa (BD Biosciences) and analyzed with FlowJo10. Quantification of caspase-1 activity was determined by the percentage of CD11c+ cells in the FLICA+ gate.

### Statistical Analysis

Data are expressed as median ± interquartile range (IQR), if not stated otherwise. Wilcoxon matched pairs signed rank test was used to compare two groups of paired data. When more than 2 groups of unpaired data were compared, Kruskal-Wallis test was performed and if significant followed by Mann-Whitney *U*-test for comparison of two specified groups as indicated. Data were analyzed with a statistical package program (GraphPad Prism 8, La Jolla, CA, USA). Differences were considered statistically significant when the *p* < 0.05.

## Results

### vWF Binds to CC in a C1q-Dependent Manner

**Figure 1:**
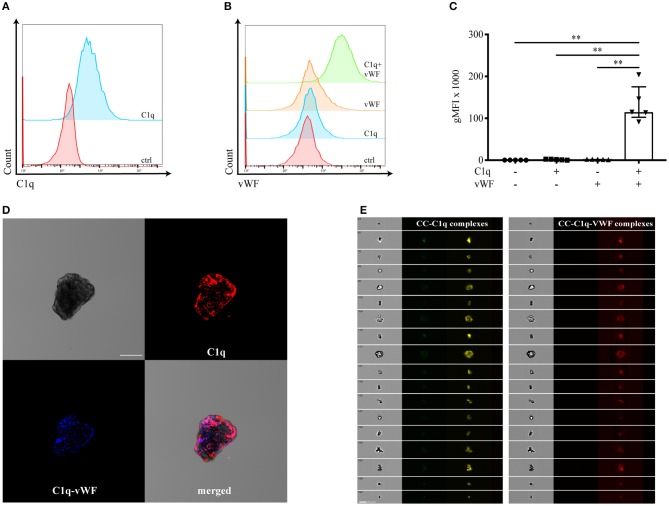
vWF binds to CC in a C1q-dependent manner. Binding of C1q and vWF to surface-bound C1q on CC was determined by **(A–C)** flow cytometry, **(D)** confocal microscopy, and **(E)** ImageStreamX. Representative flow cytometry diagrams show the binding of **(A)** C1q or **(B)** C1q-dependent binding of vWF on the surface of CC. Controls (ctrl) represent the presence of secondary antibodies only. **(C)** Flow cytometry data are shown as median gMFI with IQR (*n* = 5, Mann-Whitney *U*-test, **p < 0.01). **(D)** Confocal microscopy depicting a representative CC in brightfield, with C1q (red) or vWF (blue) in the presence of C1q bound to its surface. The merged staining patterns visualize the C1q-vWF interaction. One of three independent experiments is shown. Scale bar = 50 μm. **(E)** Fluorescent staining of CC captured by ImageStreamX for C1q (yellow) or C1q-vWF (red). One of two independent experiments is shown. Scale bar = 10 μm.

Whereas, C1q is described as a classical opsonin for a variety of DAMPs (10), the molecule has been also shown to adhere to oxidized LDL (6). In addition, Samstad et al. demonstrated C1q binding on CC after incubation with human plasma (7). Therefore, we first analyzed whether surface-bound C1q on CC secondarily enables the binding of vWF. We characterized the binding of vWF to C1q on the surface of CC by flow cytometry (Figures 1A–C), confocal microscopy (Figure 1D), and imaging flow cytometry (Figure 1E). C1q deposition on the surface of CC is shown in Figure 1A. The incubation of CC with vWF in the absence of C1q showed no vWF deposition on the CC surface (orange histogram in Figures 1B,C). Only in the presence of surface-bound C1q, vWF was enabled to bind (green histogram in Figures 1B,C). The gMFI for vWF binding in the presence of C1q was 50-fold higher compared to CC without C1q [median gMFI (IQR) of C1q+vWF: 115,000 (102,000–175,000) vs. vWF: 2,300 (1,500–2,400), *p* = 0.0079]. Furthermore, we analyzed the localization of vWF binding to CC-C1q complex. Using confocal microscopy, C1q and vWF could be visualized on CC. C1q and vWF stainings co-localized (Figure 1D). Finally, we used imaging flow cytometry to analyze a larger CC population as CC have a heterogenous structure. Again, we observed a similar staining pattern for C1q and vWF on CC (Figure 1E).

Taken together, our results demonstrate that bound C1q mediates the binding of vWF to CC, and vWF alone is not able to bind to the surface of CC.

### CC-C1q-vWF Complexes Upregulate the Surface Receptor Expression of HMDMs

**Figure 2:**
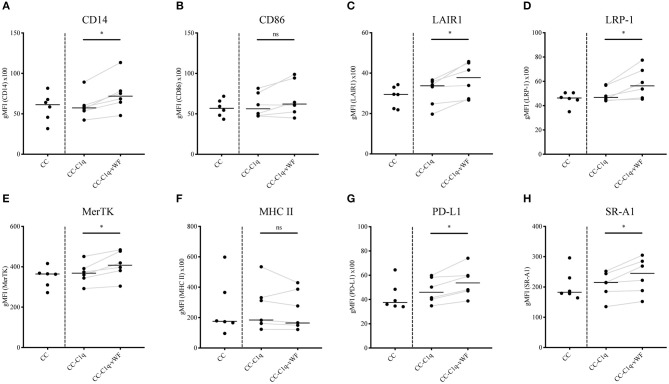
CC-C1q-vWF complexes upregulate the surface receptor expression of HMDMs. LPS-stimulated HMDMs were treated with CC, CC-C1q or CC-C1q-vWF complexes for 18 h and analyzed by flow cytometry for surface marker expression of **(A)** CD14, **(B)** CD86, **(C)** LAIR1, **(D)** LRP-1, **(E)** MerTK, **(F)** MHC II, **(G)** PD-L1, and **(H)** SR-A1. Pooled flow cytometry data are shown as gMFI with horizontal lines denoting median. Data points represent independent experiments analyzing six different healthy donors used to obtain HMDMs (Wilcoxon matched pairs signed rank test, *p < 0.05; ns, not significant).

Macrophages have a high degree of plasticity, enabling these cells to change their phenotype according to the environmental stimuli (32). In this context, C1q has been shown to elicit upregulated expression of MerTK receptor, which is involved in the process of dead cell removal, termed efferocytosis (33). Moreover, it has been described that stimulation of macrophages with C1q leads to a polarization of these cells toward an anti-inflammatory state (34). Therefore, we aimed to investigate the phenotype of HMDMs in our *in vitro* model. For this purpose, HMDMs were kept untreated or treated with CC, CC-C1q, or CC-C1q-vWF complexes for 18 h. To mimic the inflammatory milieu present in, e.g., atherosclerotic plaques (35), HMDMs were simultaneously exposed to 100 ng/ml LPS for 18 h. The phenotype was studied by analyzing the expression of surface CD14, CD86, LAIR1, LRP-1, MerTK, MHC II, PD-L1, and SR-A1 (Figures 2A–H). HMDMs treated with CC-C1q-vWF complexes significantly upregulated the expression of CD14 (*p* = 0.0312), LAIR1 (*p* = 0.0312), LRP-1 (*p* = 0.0312), MerTK (*p* = 0.0312), PD-L1 (*p* = 0.0312), and SR-A1 (*p* = 0.0312) as compared to CC-C1q complexes without vWF. In four out of six donors, CD86 expression was upregulated, while MHC II expression was downregulated in five out of six donors. Neither the median receptor expression of CD86 nor of MHC II was significantly affected. Also, CC treatment did not induce any significant changes in surface receptor expression as compared to untreated HMDMs (data not shown).

Our results demonstrate that CC-C1q-vWF complexes uniquely affect the expression of surface receptors, namely an upregulation of efferocytosis receptor MerTK, scavenger receptors LRP-1 and SR-A1 as well as CD14, LAIR1, and PD-L1.

### Phagocytosis of CC-C1q-vWF Complexes by HMDMs Is Hampered

**Figure 3:**
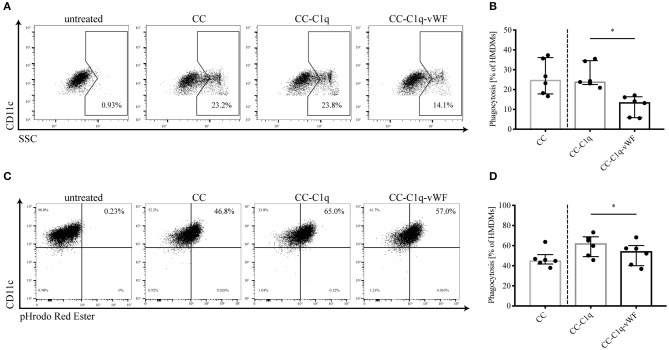
Phagocytosis of CC-C1q-vWF complexes by HMDMs is hampered. **(A,B)** HMDMs were kept untreated or treated with CC, CC-C1q or CC-C1q-vWF complexes for 18 h and analyzed by flow cytometry for the degree of phagocytosis of CC complexes. Phagocytosis was determined by the percentage of CD11c+ cells with a shift into the SSC^high^ gate. **(A)** Flow cytometry dot plots show one out of six independent experiments. **(B)** Quantification of phagocytosis for pooled data is shown. **(C,D)** HMDMs were treated with CC, CC-C1q or CC-C1q-vWF pHrodo-dyed complexes for 30 min and analyzed by flow cytometry for the degree of phagocytosis of CC complexes. Phagocytosis was determined by the percentage of CD11c+ cells with a shift into pHrodo Red Ester+ gate. **(C)** Flow cytometry dot plots show one out of six independent experiments. **(D)** Quantification of phagocytosis for pooled data is shown. Columns denote median while error bars indicate interquartile range. Data points represent independent experiments analyzing six different healthy donors used to obtain HMDMs (Wilcoxon matched pairs signed rank test, *p < 0.05).

Since C1q is involved in the processes of efferocytosis (36) as well as phagocytosis (9) and as the additional presence of vWF upregulates efferocytosis and scavenger receptors (Figure 2), we next investigated the role of C1q-vWF binding in the uptake of CC complexes by HMDMs (Figure 3). Therefore, HMDMs were kept untreated or treated with CC, CC-C1q, or CC-C1q-vWF complexes for 18 h. The phagocytosis of CC led to an increase in cell granularity, which could be determined by a shift in SSC using flow cytometry. Analyzed as control, untreated CD11c+ HMDMs did not express a SSC^high^ population. When HDMDs were treated with CC, CC-C1q, or CC-C1-vWF complexes, the cells exhibited a SSC^high^ population (Figure 3A). HMDMs showed a significant decrease in cells positive for phagocytosis after the treatment with CC-C1q-vWF complexes compared to CC-C1q complexes [median phagocytosis (IQR) in six independent donors of CC-C1q-vWF: 13.65% (5.83–16.35%) vs. CC-C1q: 24.05% (22.55–34.60%), *p* = 0.0312] (Figure 3B). To exploit the effect on early phagocytosis, we incubated CC with the pH-dependent pHrodo Red dye (Figures 3C,D). Analyzed as control, unstimulated CD11c+ HMDMs only exhibited a dim fluorescent signal for pHrodo Red. Fluorescent signal for pHrodo Red increased strongly when HMDMs were treated with pHrodo-dyed CC complexes for 30 min, due to the fusion of phagocytosed CC with the acidic lysosome of HMDMs. For the early phagocytosis, HMDMs had phagocytosed significantly less CC-C1q-vWF complexes than CC-C1q complexes [median phagocytosis (IQR) in six independent donors of CC-C1q-vWF: 54.55% (40.05–60.03%) vs. CC-C1q: 62, 40% (49.05–68.78%), *p* = 0.0312] (Figure 3D).

In summary, late as well as early phagocytosis, by HMDMs, of CC-C1q-vWF complexes is reduced as compared to CC-C1q complexes.

### CC-C1q-vWF Complexes Reduce IL-1 Cytokine Secretion of HMDMs

**Figure 4:**
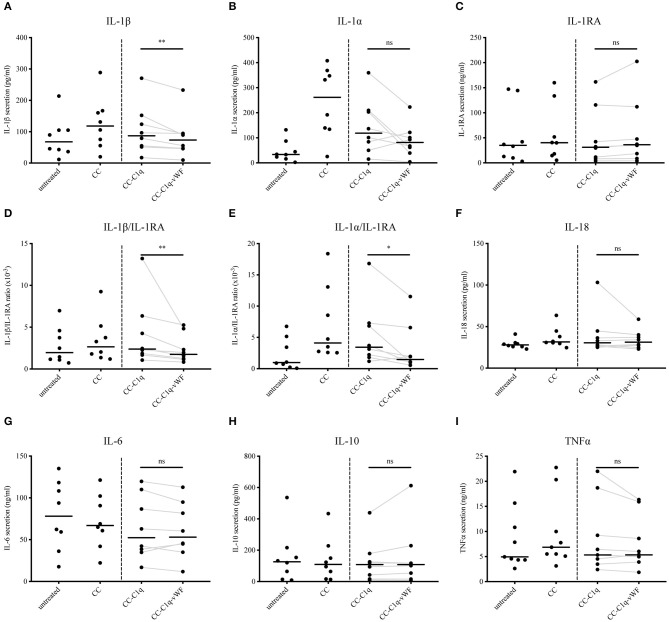
CC-C1q-vWF complexes diminish LPS-induced IL-1 secretion of HMDMs. LPS-induced HMDMs were kept untreated or treated with CC, CC-C1q, or CC-C1q-vWF complexes for 18 h. Supernatants were analyzed by ELISA for cytokine secretion. Data show median cytokine concentrations of **(A)** IL-1β, **(B)** IL-1α, **(C)** IL-1RA, **(F)** IL-18, **(G)** IL-6, **(H)** IL-10, and **(I)** TNFα levels or median ratios of **(D)** IL-1β/IL-1RA and **(E)** IL-1α/IL-1RA of pooled donors. Data points represent independent experiments analyzing eight different healthy donors used to obtain HMDMs (Wilcoxon matched pairs signed rank test, *p < 0.05; **p < 0.01; ns, not significant).

CC have been repeatedly described as capable inducers of IL-1ß secretion in human monocytes and macrophages (23). On the contrary, C1q has been shown to dampen pro-inflammatory cytokine secretion for the same cell types (34). Consequently, we next examined the effect of CC-C1q-vWF complexes on the cytokine profile of HMDMs. For this purpose, HMDMs kept untreated or treated with CC, CC-C1q, or CC-C1q-vWF complexes were stimulated with 100 ng/ml LPS for 18 h and supernatants were analyzed for the secretion of IL-1ß, IL-1α, IL-1RA, IL-18, IL-6, IL-10, and TNFα cytokine levels (Figure 4). The CC treatment induced a strong IL-1ß and IL-1α secretion by HMDMs and a moderate increase in IL-18 secretion as compared to untreated HMDMs. A robust decrease in pro-inflammatory cytokines for IL-1ß and IL-1α was observed with CC-C1q complexes, and a decreasing trend for IL-6 and TNFα secretion. The additional presence of vWF on CC-C1q complexes significantly enhanced reduction of IL-1ß secretion (*p* = 0.0078), IL-1ß/IL-1RA ratio (*p* = 0.0078), and IL-1α/IL-1RA ratio (*p* = 0.0234) compared to CC-C1q complexes alone. No other cytokines were significantly changed by vWF bound to CC-C1q complexes. Differences in cytokine secretion of HMDMs according to the treatment added were not due to differences in cell death as assessed by quantification of early and late apoptosis or necrosis (data not shown).

Taken together, our data show that IL-1ß cytokine secretion and IL-1ß/IL-1RA and IL-1α/IL-1RA ratio by HMDMs after exposure to CC-C1q complexes are diminished further in the presence of vWF. This reduction appears to be IL-1 specific.

### CC-C1q-vWF Complexes Suppress Caspase-1 Activity of HMDMs

**Figure 5:**
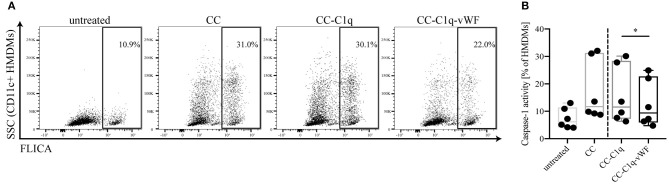
CC-C1q-vWF complexes lead to a reduced caspase-1 activity in HMDMs. HMDMs were kept untreated or treated with CC, CC-C1q, or CC-C1q-vWF complexes for 18 h and analyzed for caspase-1 activity by flow cytometry. Activity of caspase-1 was determined by the percentage of CD11c+ cells in the FLICA+ gate. **(A)** Flow cytometry dot plots show one out of six independent experiments. **(B)** Quantification of caspase-1 activity for pooled data is shown. Horizontal lines in the box plots denote median while the boxes indicate interquartile range and whiskers minimum and maximum values. Data points represent independent experiments analyzing six different healthy donors used to obtain HMDMs (Wilcoxon matched pairs signed rank test, *p < 0.05).

It is well-known that IL-1 maturation, cleavage and secretion is regulated transcriptionally as well as post-transcriptionally. While a priming signal through pattern recognition receptors is required for pro-IL-1β transcription, the maturation is dependent on the formation of the NLRP3 inflammasome and subsequent caspase-1 activation (37). Therefore, we aimed to examine whether the observed change in IL-1 cytokine secretion was the result of a preceding NLRP3 inflammasome assembly. To address this point, HMDMs were kept untreated or treated with CC, CC-C1q, or CC-C1q-vWF complexes for 18 h and the effect on caspase-1 activation was quantified with FLICA probes. Upon CC treatment, HMDMs showed a marked increase in FLICA signal, demonstrating caspase-1 activity (Figure 5A). While the presence of C1q on CC exhibited only a delicate reduction in caspase-1 activity, the additional presence of vWF significantly suppressed caspase-1 activity in HMDMs [median FLICA+ cells (IQR) in six independent donors of CC-C1q: 11.54% (7.29–28.38%) vs. CC-C1q-vWF: 9.37% (5.92–22.73%), *p* = 0.0312)] (Figure 5B).

Overall, our data show that HMDMs treated with CC-C1q-vWF complexes exhibit decreased caspase-1 activity that impacts on NLRP3 inflammasome dependent IL-1β secretion.

## Discussion

The cross-talk between the complement and the hemostatic systems is extensive and can provide synergistic benefits for the human body (38, 39). Yet, the role of many of these interplays is still unknown. In particular, even though an interplay between bound complement C1q and vWF has been demonstrated previously (17), its impact on the immune system has remained unexplored until now. In our study, we can illustrate that CC provide another physiological surface that allows a C1q-vWF interaction. Moreover, we found that the binding of vWF to bound C1q on CC is capable of modulating the immune response of macrophages by an upregulated expression of phagocytosis-mediating receptors and costimulatory receptors, a hampered phagocytosis and an enhanced suppression of pro-inflammatory cytokine secretion compared to C1q on CC alone.

Deposition of CC is described as a hallmark of atherosclerotic plaques. After recognition as DAMPs and ingestion by phagocytes, CC trigger ROS formation and lysosomal leakage with consecutive NLRP3 inflammasome assembly, caspase-1 generation and IL-1β secretion (24). IL-1β secretion leads to further recruitment of phagocytes by an amplification loop in a concerted action with other pro-inflammatory cytokines and chemokines (40). Phagocytes, in particular macrophages, also are responsible for the essential function of recycling LDL and cholesterol in the periphery, but can develop into lipid-laden macrophage-derived foam cells during the course of the disease when their recycling capacity is overwhelmed. First, those foam cells can become apoptotic due to various stimuli, such as prolonged endoplasmic reticulum stress. Second, apoptotic cells that are insufficiently cleared (as occurring in advanced lesions due to defective efferocytosis), advance into cellular necrosis, in turn contributing to the formation of the necrotic core (41). Consequently, enhanced ingestion of LDL and CC fuels foam cell development, which is thought to be detrimental in later stages of atherosclerosis (42). Hence, the conclusion that CC induce arterial inflammation and destabilization of atherosclerotic plaques seems to be plausible (43). The complement molecule C1q can be considered as a double-edged sword in the context of atherosclerosis. Previous studies showed that the clearance of oxidized LDL and modified LDL is enhanced by binding of C1q (6), but simultaneously leads to a polarization of macrophages toward an anti-inflammatory phenotype through a reduction in pro-inflammatory cytokine secretion (44). In addition, C1q induces mRNA transcription of cholesterol efflux transporters (6). In contrast to these atheroprotective traits, C1q was demonstrated to be present on CC from human plasma (45) and found to be complexed to ApoE in human arteries (46), where it enables complement activation and thus contributes to atheroprogression (47, 48).

With regard to vWF, a number of studies in vWF-deficient animals and in patients suffering from Von Willebrand disease have been performed. Several of those animal studies (29) as well as human studies (49, 50) suggest atheroprogressive effects of vWF. Therefore, one could hypothesize disadvantageous consequences for the additional presence of vWF on CC-C1q complexes on macrophages. However, our findings unexpectedly point to a beneficial effect of vWF in the context of phagocytosis of CC by macrophages, and suggest that the role of vWF in atherosclerosis might be intricate and requires further investigation.

Previously, C1q and vWF have been regarded as separately acting molecules. Here, we identified not only a complex formation of C1q bound to the surface of CC but also the subsequent binding of vWF. Moreover, the treatment of HMDMs with CC-C1q-vWF complexes results in an upregulated expression of surface receptors of efferocytosis (MerTK), scavenger receptors (LRP-1 and SR-A1) as well as CD14, LAIR1, and PD-L1 compared to CC-C1q complexes alone. Studies investigating the role of the phagocytosis-mediating receptors MerTK and LRP-1 indicate atheroprotective features (51, 52), whereas the role of SR-A1 in cardiovascular disease is still controversial [reviewed by Ben et al. (53)]. Additionally, LAIR1 was described to have beneficial effects on foam cell formation (54). Therefore, we next sought to determine the effect on the phagocytic capacity of HMDMs. Interestingly, the presence of vWF on CC-C1q complexes strongly diminished the late as well as early phagocytosis of CC by HMDMs, hereby reversing the effect of C1q alone. A possible explanation for this unexpected finding could be that the upregulated expression of phagocytosis-mediating receptors is representing a reinforcing feedback loop that is triggered in order to compensate for the decreased ingestion of CC-C1q-vWF complexes. Last, our data illustrate a significant decrease in IL-1 cytokine secretion by HMDMs when treated with CC-C1q-vWF complexes compared to CC-C1q complexes without vWF. The clinical significance of IL-1 in cardiovascular disease was demonstrated by the anti-IL-1beta antibody Canakinumab Antiinflammatory Thrombosis Outcome Study (CANTOS) (55). Thus, a reduction in phagocytosis and inflammation could retard plaque progression (56, 57).

One limitation of our study is its *in vitro* character, since the *in vivo* situation in humans is likely to be more complex. C1q's role in human atherosclerosis is supported by studies that have shown C1q expression in atherosclerotic carotid arteries of patients (58–60) and therefore underlines the relevance of our results. Whereas the majority of C1q is non-covalently bound with serine proteases C1r and C1s to form the C1 complex in plasma and whole blood, free C1q is more prevalent in tissues where it is locally synthesized mainly by macrophages and dendritic cells (61). Furthermore, it has been demonstrated that vWF binds to a cryptic epitope of C1q, which is only exposed when C1q is surface-bound, while binding of vWF to surface-bound C1 was much weaker (17). Hence, we assume that the C1q-vWF interaction, especially on CC, primarily occurs in tissue, such as arteries of atherosclerotic patients. Nevertheless, further investigation on the occurrence of CC-C1q-vWF complexes in human atherosclerosis is needed.

Second, *in vivo*, shear stress is necessary to unfold the full functional potential of vWF (62). In our study however, permanent shear stress was not applied, since the physiological occurrence of shear stress would rather reflect the situation during plaque rupture resulting from continuous blood flow but not that inside the plaque itself.

Lastly, alternative ways can be envisaged by which the C1q-vWF interaction, in the form of CC-C1q-vWF complexes, might exert its effect on HMDMs. One of the ways could be partial steric shielding of the C1q molecule by vWF, weakening the effects of C1q (e.g., Figure 3). Another way could be an intrinsic effect of vWF (e.g., Figures 2, 4). Future studies will have to explore the potential ways responsible for the overall impact of CC-C1q-vWF complexes. In addition, since the mutual interactions between complement and hemostatic systems *in vivo* are likely to be more complex, our *in vitro* model will have to be developed further in order to approach a physiological setting. Recently, Gravastrand et al. have described that CC induce complement-dependent activation of hemostasis (63). In our group, we have observed that complement activation remains unaffected by the presence of vWF (64). Hence, downstream complement components, such as C4 and C3, shall be implemented into our system and its effect on HMDMs in the additional presence of platelets addressed in the future.

In conclusion, with this study, we provide new insights into an emerging cross-talk between C1q and hemostasis-initiating vWF. Our findings reveal that binding of vWF to C1q on CC regulates the immune response of HMDMs. We show that CC-C1q-vWF complexes provoke a hampered phagocytosis together with an accompanied reduction of IL-1 cytokine secretion by macrophages that could prove favorable for retarding foam cell formation and decelerating plaque progression.

## Data Availability Statement

All datasets generated for this study are included in the article/supplementary material.

## Author Contributions

CD designed and performed the study, collected and analyzed data, and wrote the manuscript. ST and MT designed and supervised the study, and critically revised the manuscript.

### Conflict of Interest

The authors declare that the research was conducted in the absence of any commercial or financial relationships that could be construed as a potential conflict of interest.
